# Procedure Increasing the Accuracy of Modelling and the Manufacturing of Surgical Templates with the Use of 3D Printing Techniques, Applied in Planning the Procedures of Reconstruction of the Mandible

**DOI:** 10.3390/jcm10235525

**Published:** 2021-11-25

**Authors:** Paweł Turek, Paweł Pakla, Grzegorz Budzik, Bogumił Lewandowski, Łukasz Przeszłowski, Tomasz Dziubek, Sławomir Wolski, Jan Frańczak

**Affiliations:** 1 Faculty of Mechanical Engineering and Aeronautics, Rzeszów University of Technology, 35-959 Rzeszów, Poland; gbudzik@prz.edu.pl (G.B.); lprzeszl@prz.edu.pl (Ł.P.); tdziubek@prz.edu.pl (T.D.); 2 Department of Maxillofacial Surgery, Fryderyk Chopin Clinical Voivodeship Hospital No.1 in Rzeszów, 35-055 Rzeszów, Poland; pawel.pakla@gmail.com (P.P.); boglewandowski@wp.pl (B.L.); janek.franczak@gmail.com (J.F.); 3 Collegium Medicum, University of Rzeszów, 35-315 Rzeszów, Poland; 4 Faculty of Mathematics and Applied Physics, Rzeszów University of Technology, 35-959 Rzeszów, Poland; wolan@prz.edu.pl

**Keywords:** medical engineering, reconstructive surgery, mechanical engineering, additive manufacturing, accuracy, surgical models, polymer material, fibular free flap, virtual surgical planning, optical coordinate measurement system

## Abstract

The application of anatomical models and surgical templates in maxillofacial surgery allows, among other benefits, the increase of precision and the shortening of the operation time. Insufficiently precise anastomosis of the broken parts of the mandible may adversely affect the functioning of this organ. Applying the modern mechanical engineering methods, including computer-aided design methods (CAD), reverse engineering (RE), and rapid prototyping (RP), a procedure used to shorten the data processing time and increase the accuracy of modelling anatomical structures and the surgical templates with the use of 3D printing techniques was developed. The basis for developing and testing this procedure was the medical imaging data DICOM of patients treated at the Maxillofacial Surgery Clinic of the Fryderyk Chopin Provincial Clinical Hospital in Rzeszów. The patients were operated on because of malignant tumours of the floor of the oral cavity and the necrosis of the mandibular corpus, requiring an extensive resection of the soft tissues and resection of the mandible. Familiarity with and the implementation of the developed procedure allowed doctors to plan the operation precisely and prepare the surgical templates and tools in terms of the expected accuracy of the procedures. The models obtained based on this procedure shortened the operation time and increased the accuracy of performance, which accelerated the patient’s rehabilitation in the further course of events.

## 1. Introduction

The rapid development that has been observed in recent years in the area of tools and information systems in mechanical engineering, including the development of methods of rapid prototyping (RP) [1,2] and reverse engineering (RE) [3,4], can be successfully used in medicine in order to optimize and improve the quality of therapy [5]. Obtained based on medical imaging data, virtual and real three-dimensional (3D) models find their application according to the literature analysis in such specialties as maxillofacial surgery and dentistry (58.3%) [6,7,8,9] and orthopaedics (23.7%) [10,11,12,13]. Other areas include neurosurgery [14], oncology [15], plastic surgery [16], cardiology [17], laryngology [18], dermatology [19], and pulmonology [20]. In the areas mentioned earlier, both anatomical models for planning procedures and surgical templates are usually used to increase precision and shorten the operation time [21,22,23,24]. The development of these procedures is a complex and interdisciplinary process. It requires extensive experience, medical and technical knowledge (particularly in the fields of anatomy, radiology, mechanics, and biomedical engineering), knowledge of modern computer-aided design methods (CAD), RP, and RE.

Procedures related to the reconstruction of the facial skeleton [25,26,27], performed in patients requiring extensive resection of soft tissues and bones due to malignant tumours of the floor of the mouth and mandibular necrosis [28,29,30], are particularly demanding in terms of precision and the risk of complications. This work focuses on developing a procedure for reconstructing the mandible, which is the most specific bone structure in the stomatognathic system. It is one moving bone subjected to multidirectional dynamic loads in the masticatory organ [31]. So far, titanium reconstructive plates have been used the most frequently to reconstruct the mandible continuity [32,33]. Currently, their usage should be limited in patients with systemic burdens. They are used in patients with contraindications to extensive reconstructive procedures, which last many hours. The introduction of new techniques based on microsurgical anastomosis of vascularized free tissue grafts containing bone elements to restore the continuity of the mandible using the fibula [34,35] or the iliac plate bone [36,37] is currently the gold standard in maxillofacial oncology. Thus, the titanium plates were limited to an auxiliary function ensuring the stabilization of the graft during the healing period. Methods known from the technical sciences are now successfully used to improve the therapeutic process within the lower face. Models of anatomical structures made with the help of RE/RP techniques facilitate the procedure mainly due to earlier adjustment of the plates or titanium mesh to the model [38,39,40,41]. However, these stereolithographic models are not able to fully transfer the virtual plan to the real surgery. Now, due to the continuous development of computer-aided manufacturing (CAM) and RP methods, manufacturing cutting guides and plates designed to follow the contour of the patient’s bone is also possible [42,43,44]. Templates for transferring a virtual surgery plan to a real surgical procedure made using the selective laser melting (SLM) [45] or direct metal laser melting (DMLS) [46] additive techniques. Most often, implant models are made of the pre-alloyed Ti6AlIV4 alloy [47,48]. This material has excellent mechanical properties, corrosion resistance, and good biocompatibility. The use of CAD/CAM methods will increase precision and reduce the procedure’s time [49,50,51].

One of the key parameters of the anatomical model or surgical template is that it should be accurately made. The digitization stage has the greatest impact on the accuracy of mapping the geometry of anatomical structures [52,53,54]—this way, the obtained data are transformed into a three-dimensional model. At this stage, the process of segmentation plays the most important role (including the applied method and the parameters extracting the anatomical structure from the digital imaging and communications in medicine (DICOM) data) and the geometry reconstruction process used in various methods (e.g., planar contour or voxel-based methods) [55,56]. The accuracy of reconstructing the geometry of the anatomical structure is also influenced by the selection of the manufacturing method and parameters [57,58,59,60]. Despite the numerous scientific studies that have been carried out in recent years discussing the use of 3D modelling methods which enable the reconstruction of the geometry of anatomical structures models for the implementation of implants or surgical templates, there is no study on a systematic procedure that allows for increasing and controlling their accuracy, particularly for the area of the lower face part, i.e., the mandible in which its parabolic shape should be maintained. Unintentional inaccuracies or mistakes can often arise at every stage, from the digitization process through the reconstruction of the geometry of the anatomical structure to the implementation of the physical model. This can significantly affect the accuracy and precision of the surgical procedure. The development of these procedures can help prepare templates and surgical tools, ensure precise execution of planned operations, shorten the time of the procedure, reduce blood loss, use of anaesthetic drugs, and reduce postoperative complications (thus resulting in faster recovery).

## 2. Materials and Methods

**Figure 1:**
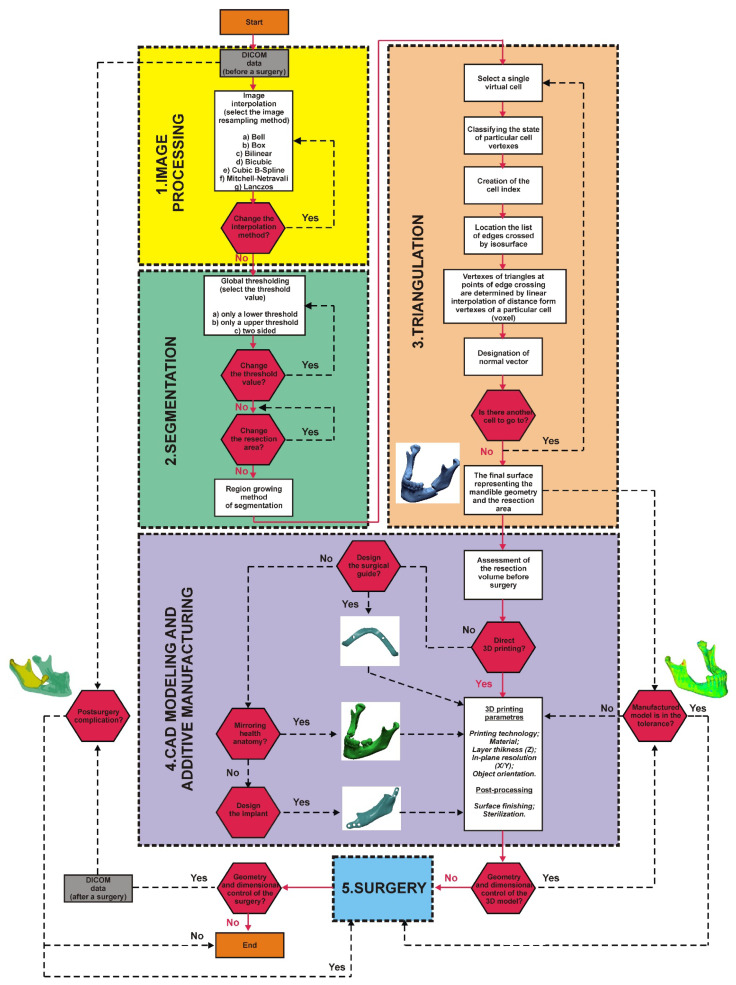
Procedure applied in the research process.

As part of the cooperation agreement concluded in 2018 between the Fryderyk Chopin Provincial Clinical Hospital No. 1 in the Rzeszów–Maxillofacial Surgery Clinic and the Rzeszów University of Technology, studies were carried out according to the guidelines of the Declaration of Helsinki and approved by the Bioethics Committee of Medical Board in Rzeszow (62/B//2018) in a group of 14 patients. This group included 12 patients treated and operated on for oral squamous cell carcinoma of the floor of the mouth with malignant infiltration of the mandibular body and 2 patients treated and operated on due to necrosis of the mandibular corpus. All patients in this group who gave informed consent for the operation and the use of the data for research required extensive resection of the primary malignant tumour, mandibular resection, and radiation necrosis of the mandible; the resection of healthy tissues was also required. There were nine women and five men in the group of 14 patients. Patients over 55 years of age were selected for the research group because, in this group of patients, the highest percentage of people requires surgery (which is often life-saving). In addition, the motivation for choosing patients over 55 resulted from problems with the precise segmentation of bone tissues in the three-dimensional modelling of anatomical structures. Based on the selected group of patients, a procedure was developed that allows to shorten the time of data processing and increase the accuracy of modelling and the production of surgical templates using 3D printing techniques, in order to increase the precision of procedures which allow the reconstruction of the anatomical continuity of the mandible. The diagram of the procedure algorithm is presented in Figure 1. The procedure was presented based on one of the 14 patients treated in the research process.

### 2.1. Procedure of the Reconstruction of Geometry and Modelling Templates

**Figure 2:**
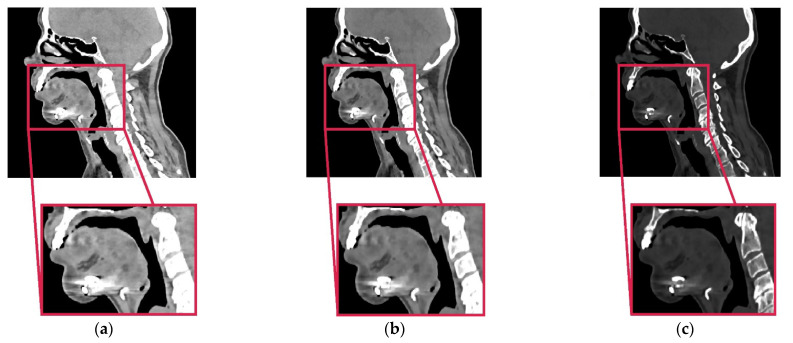
DICOM data: (**a**) without the application of procedure; (**b**) with the application of the procedure using the noise reduction filter—improve contrast resolution; (**c**) with the application of the procedure using Lanczos interpolation method—improve spatial resolution.

In the imaging of the facial part of the skull in the Fryderyk Chopin Provincial Clinical Hospital No. 1 named after Fryderyk Chopin in Rzeszów, a scanning protocol is observed (Tubesettings: 100 kV, 158 eff. mAs; Colimation: 32 × 1.2 mm; Aquiredslicewidth 1.2 mm; Reconstructedslicewidth 1.5 mm; Matrixsize: 512 × 512). The multi-slice tomograph Somatom Definition AS+ (Siemens Medical Solutions, Forchheim, Germany) was used to carry out the research. The obtained DICOM data are characterized by high special resolution due to anisotropic voxel structure of volumetric data (0.4 mm × 0.4 mm × 1.5 mm). This voxel anisotropy together with relatively low resolution of a standard protocol generates partial volume artifact, which significantly hinders the process of segmentation. It blurs the margins of the object and is the source of contour discontinuities of the reconstructed anatomical structure (Figure 2a). The impact of partial volume artifact can be limited via the implementation of high definition protocols of reconstruction and thin layers (low collimation value) [61,62]. However, the approved measurement protocols do not allow too much interference in determined diagnostic parameters. This is due to the paramount requirement of protection of health by limiting the patient’s exposure to ionizing radiation. In order to reduce the impact of the mentioned factors, a procedure was undertaken to increase special resolution on the previously collected DICOM data. For this purpose, an image interpolation process was implemented [63,64].

Image interpolation is a term used for image processing, but it is often used with different terminologies in literature such as image scaling, image resampling, and image resize. The resampling operation does not change the coordinate type recalculating the data according to this resizing. In the case of DICOM data in the research was transformed into a 0.4 mm × 0.4 mm × 0.4 mm voxel size. Voxel-based data objects such as 3D images may be resampled onto a new grid. This can be done using several interpolation filters (they differ in their quality and computational effort) by either taking the original bounding box or using an enlarged one (which encloses the complete transformed data set). Based on the research concerning the accuracy of the model geometry mapping, the Lanczos method works best. Until now, the disadvantage of this solution was the significant increase in the size of the volumetric data, which required more memory and resources for rendering. Due to the rapid development of computerized systems, this problem no longer exists. The segmentation process was carried out on the data digitally processed this way. This process consisted of two stages. The first one defined the threshold which had been taken into account when the process of extracting the contour of the bone structure of the mandible from DICOM data was performed. Most often, this process requires only defining the lower segmentation threshold [55]. On the basis of the averaged results obtained from 14 analysed patients over 55 years of age, the value for the implemented procedure was set at 200 HU. The process also takes into account that, in the case of diagnostic data containing metallic components, an upper segmentation threshold is also defined, which allows the segmented bone structure to be separated from, for example, a titanium plate. Such a situation takes place during the segmentation of anatomical structures carried out on the DICOM data obtained after a surgical procedure. The average value of the upper segmentation threshold for the implemented procedure was set at approximately 1700 HU. This value could be more accurately estimated through the use of the procedure. Without it, it would be difficult to define the boundary between the bone tissue and the surgical plate (Figure 2a). The after-surgical DICOM data processing procedure includes an additional step compared to the before-surgical processing. Because there are noises in 2D images resulting from a titanium plate, an additional digital filtering process is performed. A minimum noise reduction filter was used for this purpose. The filtered image was obtained by applying the convolution operation, i.e., the multiplication of two frequency-domain transforms, i.e., the image and filter transform. The convolution operation calculates a new pixel value in an image based on the values of the adjacent pixels. Thanks to the filtration process, the noise was partially removed from the image, and the area of tissue connection with the implant was more emphasized (Figure 2b). Thus, this procedure partially increased the contrast resolution of DICOM data. The next stage of the procedure was to perform the interpolation process using the Lanczos method, just like in the case of DICOM data processing before the surgery. The image interpolation process allowed for the determination of additional pixels with their value based on the intensity of adjacent pixels, increasing the spatial resolution of the DICOM data (Figure 2c). The resampling method also partially minimized the occurrence of the metallic artifact that came from the surgical plate and separate it more efficiently from the bone tissue.

**Figure 3:**
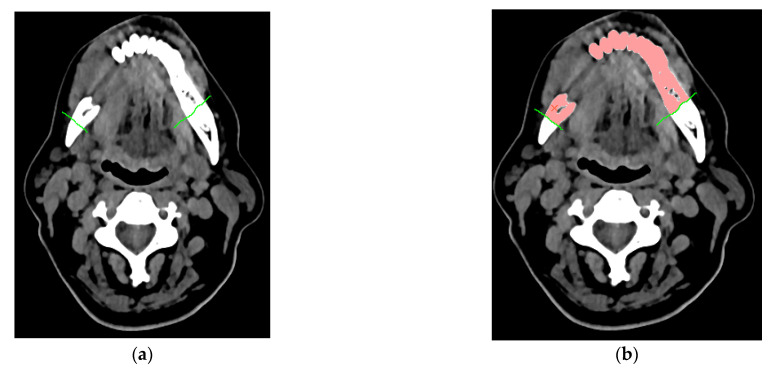
Determining the resection area: (**a**) Limiting the area with the use of segmentation curves; (**b**) separations of the area with use of region growing method.

In the case of specifying the area to be resected, the procedure additionally includes the determination of virtual segmentation curves, defined on the DICOM data (Figure 3a). They additionally enable the separation of the resected mandibular bone from the dental crowns. Thus, it is possible to estimate the volume of the resected area more accurately. This is important when the process of collecting free tissue grafts containing a bone element to restore the continuity of the mandible is expected. After introducing the initial parameters and optionally the virtual curves, the last step of the segmentation process involves the application of the region growing method. It belongs to the area method group. As a result of the application of the region growing method [65], it is possible to classify all pixels with a similar shade of grey to the triangulation process and assign them to one group that defines the entire mandible structure or the resected area (Figure 3b).

**Figure 4:**
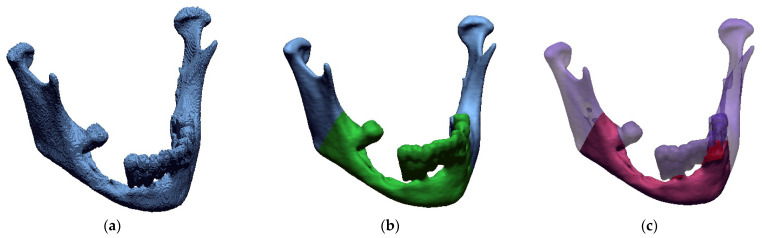
3D visualization of the geometry of the mandible: (**a**) Without the application of the procedure; (**b**) with application of the procedure together with determining resection area; (**c**) with application of the procedure in terms of estimating the volume of the resection area.

The isosurface method was used to visualize the spatial model of the anatomical structure in the triangulation process. It is based on the Marching Cubes algorithm [66,67]. This method consists in dividing the space into a series of cubes that can span one or more voxels. Then, the nodes of particular designated cubes are checked for the defined iso-value. Depending on whether the value of the node is greater or lesser, polygons corresponding to the isosurface passing between these points are inserted in the place of the cube. Eventually, a three-dimensional model representing the geometry of the mandible is obtained in the triangulation process. If the procedure is not implemented, the three-dimensional representation of the model includes a stair-step artifact [61,62,68], which is created as a result of the occurrence of partial volume artifact on 2D images (Figure 4a). Thanks to the implementation of the procedure, the impact of these artifacts was significantly reduced, which increased the accuracy of the geometry reconstruction, avoided time-consuming processes of model surface treatment before the 3D printing process, (Figure 4b) and allowed for more precise estimation of the resection area volume (Figure 4c).

**Figure 5:**
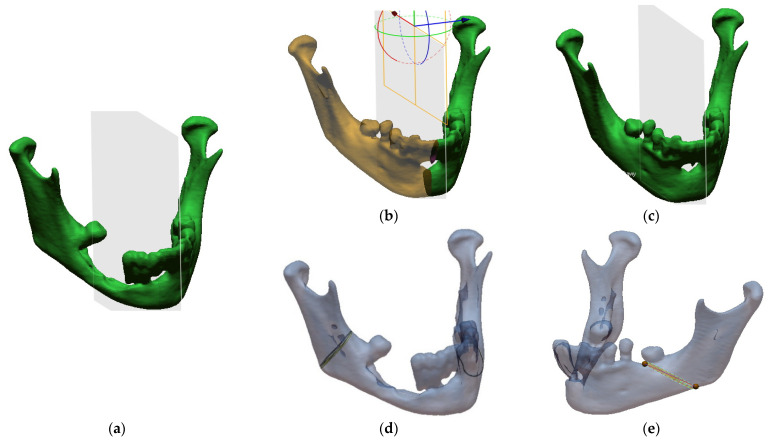
Modelling of surgical templates: (**a**) Loaded anatomical model of the mandible; (**b**) mirror image of the health part of the mandible onto the pathological part; (**c**) ready template for printing; (**d**) marked resection site from the right side; (**e**) marked resection site from the left side.

It often happens that the pathological area is significantly deformed and there are difficulties in adjusting the reconstruction plate or determining the resection place during the procedure [69,70]. Therefore, in order to have a reconstructed model of the mandible, computer aided design (CAD) procedures were applied to prepare ready-made surgical templates for a specific procedure. First, the model was loaded (Figure 5a), then a part of the healthy mandible area was mirrored onto the pathological part in relation to the YZ plane (Figure 5b). In the end, a model was obtained that allowed the titanium splint to be bent from the pathological side when planning the procedure (Figure 5c). Additionally, the sites of the mandible intersection were marked on the surface of the model reconstructed from DICOM data (Figure 5a) by defining curves. They were made on the right side (Figure 5d) and on the left side (Figure 5e).

### 2.2. Printing Models and Surgical Templates

**Figure 6:**
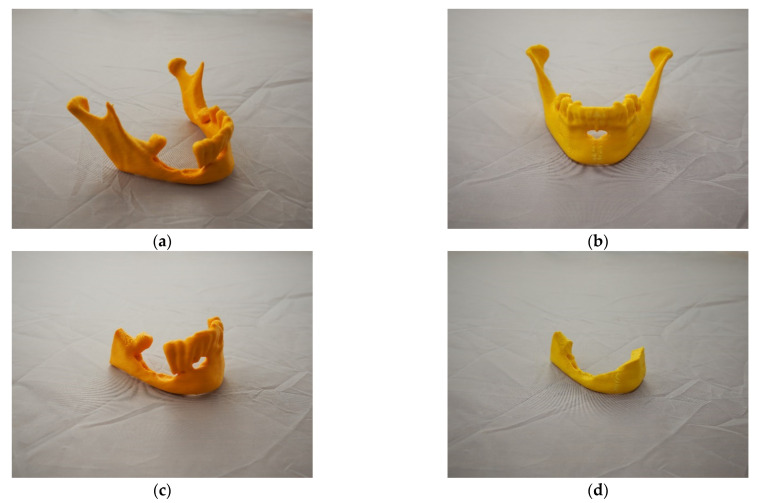
Manufacturing the models with use of 3D printing techniques: (**a**) The model of the mandible-A; (**b**) model representing mirror of the health part of the mandible onto the pathological part-B; (**c**) resection area-C; (**d**) model serving for estimating the volume of the resected bone tissue-D.

In order to make physical models, the 3D printing technique fused filament fabrication (FFF) was used. This method is equivalent to the fused deposition method (FDM) method. The creation process consists in melting the filament in a heated head, and then it is applied to the worktable. Once the first layer is placed, more layers are added to the object until the entire model is printed [71,72]. In the process of making physical models, an anatomical model of the mandible with marked resection sites (Figure 6a), a model representing mirror of the health part of the mandible onto the pathological part (Figure 6b), a resected area (Figure 6c), and a model serving for estimating the volume of the resected bone tissue (Figure 6d) were printed.

**Figure 7:**
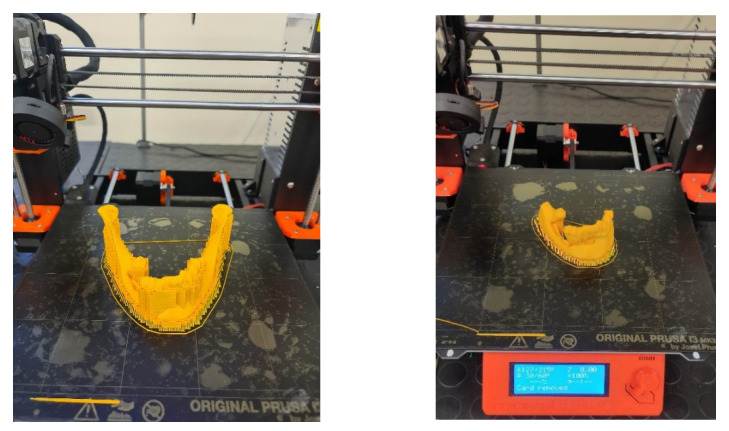
Orientation of models in printer space.

All models were made on a Prusa MK3s printer. PLA poly (lactide) material was used for printing. It is one of the main biodegradable polymers used, among others, in medicine (dental implants) [73]. This material is characterized by adequate tensile strength and stiffness. In order to increase the accuracy of the models’ printing, the smallest layer thickness (0.15 mm) was used, which could be applied by the selected system. Additionally, each model was oriented during printing so that the lateral surface of the mandible to which titanium plates are most often fitted was made along the Z axis. This procedure increased the accuracy of the surface in this area of the mandible (Figure 7).

## 3. Results

**Figure 8:**
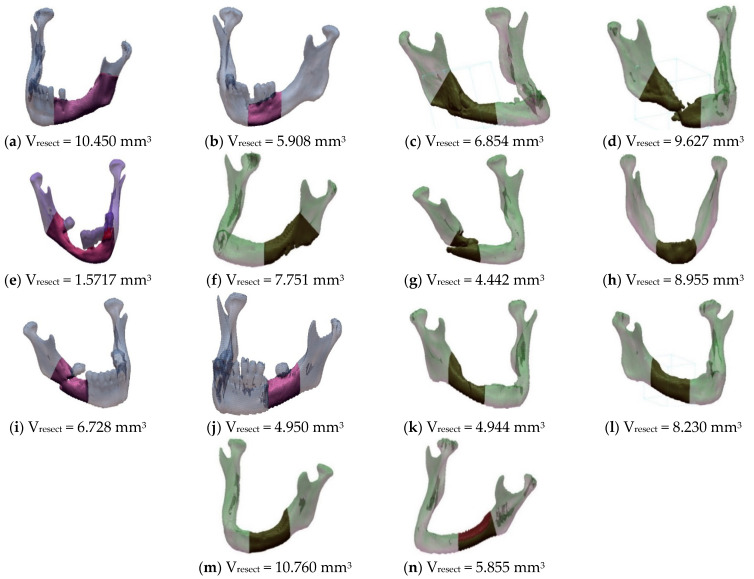
Marked resection areas with the estimated volume.

Using the functions of the Geomagic software, the volume of the resected area (V_resect_) was determined for each of the 14 examined patients (Figure 8).

**Figure 9:**
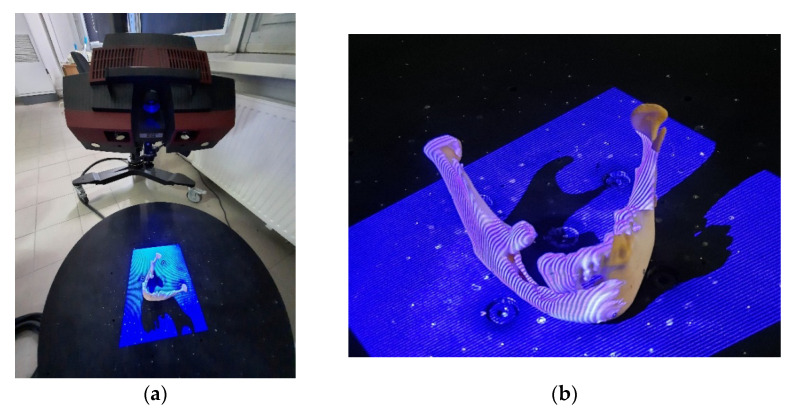
Process of the assessment of the accuracy of manufacturing models: (**a**) Atos II Triple Scan; (**b**) model during the measurement.

The models were controlled for accuracy before each planned surgical procedure. In the verification process, an automated measuring station was used, equipped with the Atos II Triple Scan (by GOM) blue light structured scanner and a rotary table [74,75] (Figure 9a). During the measurement, a specific raster (or their sequence) is projected onto the surface of the measured object (Figure 9b). The image of the deformed raster on the surface of the object is subjected to computer analysis, which results in a three-dimensional representation of the geometry of the measured object. During the tests, a procedure minimizing measurement errors was taken into account.

**Figure 10:**
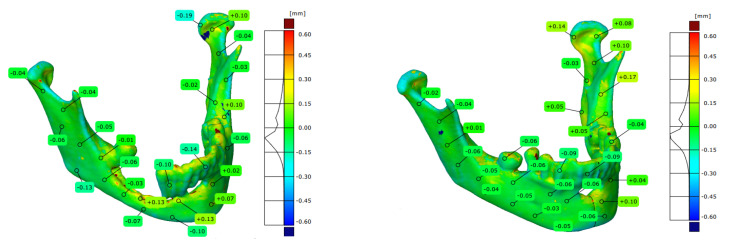

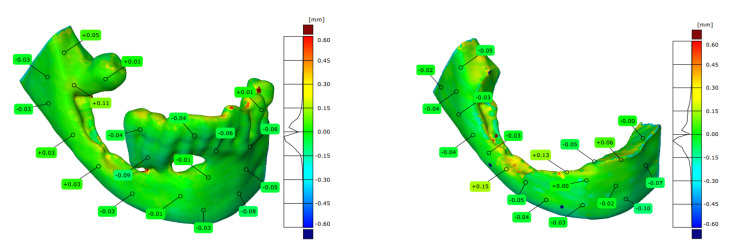
Reports presenting the accuracy of manufacturing models.

The analysis of the obtained results was carried out using the GOM Inspect software. The adjustment of the nominal model obtained at the RE/CAD design stage and the reference model created at the measurement stage using the Atos II Triple Scan optical system was carried out using the BestFit method with an accuracy of 0.001 mm. The accuracy assessment of the models was presented in the form of three-dimensional deviation maps (Figure 10).

On the basis of the presented procedure, the surgical procedure and the reconstruction of post-resection bone defects were planned. As an example of the use of the described procedure, the observation of a patient who has recently been treated in the clinic is presented below.

### Own Observation

**Figure 11:**
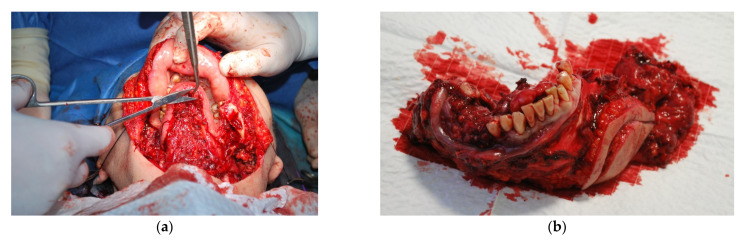
Performing the surgical procedure within the area of the mandible: (**a**) resection of the part of the mandible in particular places; (**b**) resected area obtained in the surgical process.

The patient P.W., 59 years of age, was referred due to a painful ulceration of the lower gingiva and the floor of the mouth on the right side, partially spreading to the left side, and enlarged lymph nodes on both sides of the neck and a neoplastic infiltration of the submandibular skin on the left side. After performing imaging diagnostics (CT and MRI) and taking a section from the ulcer, the patient was diagnosed with squamous cell carcinoma covering the floor of the mouth on the right side, partially on the left side of the infiltrating body and branches of the mandible with bilateral metastases to the lymph nodes in the neck and a neoplastic infiltration of the submandibular skin on the left side (T_4a_N_3b_M_o_). The patient was qualified for surgery, for which she gave her informed consent and the use of data in scientific publications. The operation consisted in removing the tumour of the lower gingiva and the floor of the oral cavity with partial resection of the mandibular branch and body on the right side and the mandibular body on the left side, bilateral removal of neck lymph nodes groups I–V with non-lymphatic structures: internal jugular vein, accessory nerve, and the sternocleidomastoid muscle. The tumour-infiltrated skin of the left submandibular area was also removed in the tissue complex. Reconstruction of the post-reaction cavity tissue defect and restoration of the continuity of the mandible was planned using a vascularized free bone-tissue flap taken from the left fibula. Adjuvant radio-chemotherapy is scheduled after the surgery. Moreover, on the basis of computed tomography, models for the planned surgical procedure were printed based on the proposed procedure (Figure 6). The model with marked bone resection sites (Figure 6a) allowed for precise determination of the places of the mandibular intersection during surgery (Figure 11a). The surgical procedure was uneventful according to the oncological protocol. The resected part (Figure 11b) of the mandible was then compared with the model planned before the procedure (Figure 6c).

**Figure 12:**
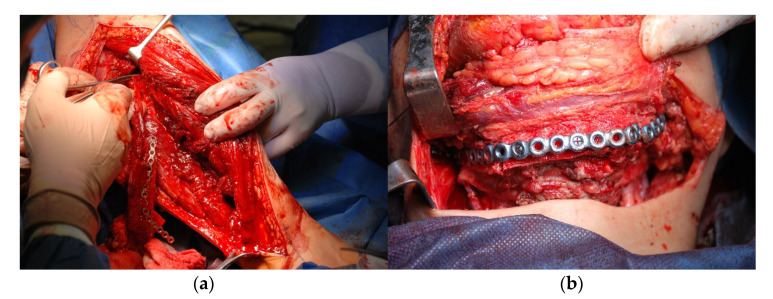
Reconstructing the continuity of the body and branches: (**a**) sampling bone tissue from the fibula; (**b**) anastomosis of the sampled bone parts with the titanium splint.

The post-resection defect was reconstructed using a previously bent titanium plate and the fibula (Figure 12a,b), which was divided into three sections to recreate the natural curvature of the mandible. Thanks to the preparation of the model illustrating the resection area (Figure 6d), it was possible to assess the bone tissue required for the extraction of the fibula more accurately.

**Figure 13:**
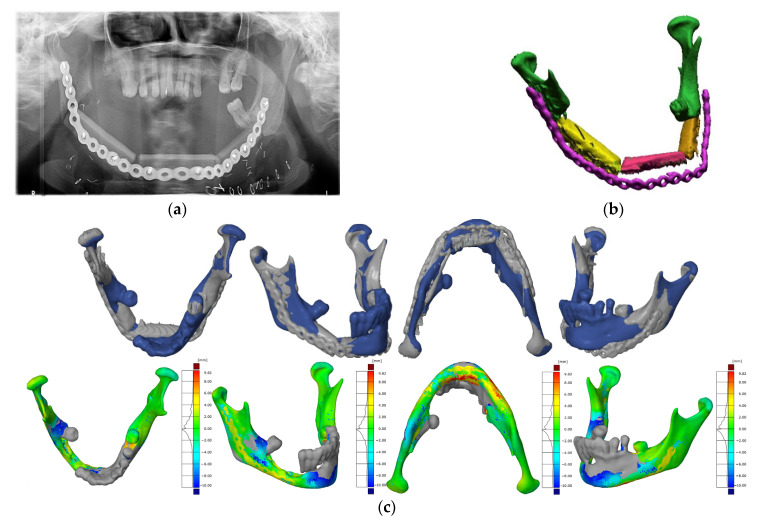
Performing diagnostics after the procedure: (**a**) panoramic radiograph; (**b**) digital reconstruction of the area of the mandible with the separated titanium splint; (**c**) analysis of the accuracy of reconstructing the anatomical continuity of the mandible.

Proper blood supply to the flap was possible due to the microvascular anastomosis of the flap artery and vein with the vessels of the neck. The loss of the floor of the mouth tissue on the right side and the loss of the neck skin on the left side were reconstructed using two skin islands of the sagittal flap. The wound of the left lower leg was sutured in layers in the proximal part. In the distal part, due to the significant tissue tension, a slow split-thickness skin graft was used. The lower limb was immobilized with a splint for 7 days. In the early postoperative period, significant swelling of the lower face was observed. Due to the bilateral cutting of the peripheral branches of the facial nerve during resection and postoperative oedema, the patient was unable to close the oral cavity. The occlusal contacts on the molars on the left side were normal. The postoperative wounds healed properly. The hypoesthesia of the skin on the lower leg and foot was denied by the patient. There were no walking disturbances. The patient was then rehabilitated regarding movement, facial movements, breathing, and swallowing. When the swelling of the face had subsided, the patient was able to close the mouth at rest. On day 56 after surgery, the patient started adjuvant radiochemotherapy. The patient underwent a control CT and OPS examination 6 months after the surgery (Figure 13a,b) and a comparative analysis was carried out after the surgery (Figure 13c).

## 4. Discussion

The specialist literature presents a lot of scientific studies regarding the use of spatial modelling methods, enabling the preparation of models of anatomical structures, surgical templates or implants [6,10,15,22,76,77]. The systematized procedure used in the article is an exception to the studies presented so far. It allows to increase and control the accuracy of creating models of anatomical structures and surgical templates within the mandible. The digitization stage particularly affects the accuracy of mapping the geometry of anatomical structures [52,53,54]. Diagnostic imaging protocols do not allow too much interference in the established diagnostic parameters. This is due to the overriding need to protect the patient against ionizing radiation. The use of the image interpolation process [56,57] in the procedure significantly increased the spatial resolution of DICOM data at the stage of numerical data processing. Thus, it allowed for a more precise analysis of DICOM data on the level of 2D images and 3D reconstruction. At the stage of segmentation, it is important to choose the right values for segmentation thresholds [53,55,56]. This process requires a lot of experience. Determining wrong values can significantly change the volume and the accuracy of mapping the shape of the geometry of the extracted anatomical structure from the DICOM data. Currently, a lot of research is being carried out in this area. Establishing averaged segmentation thresholds in the procedure not only allowed for the dimensional and shape improvement of the reconstructed geometries, but also a significant acceleration of the CAD modelling process of surgical templates. The triangulation process used in the procedure, carried out using the Marching Cubes method [66,67], made it possible to obtain a visualization of three-dimensional geometry. The main problem at this stage of numerical data processing is usually the time-consuming edition of the model surface in order to adapt it to the 3D printing process [53,56]. The algorithm used in the article allows for the direct generation of three-dimensional geometry, the surface of which does not require additional corrections before the printing process. The use of the smallest layer thickness in the printing process and the appropriate orientation of the model in the printer space allowed to increase the accuracy of the printout.

**Figure 14:**
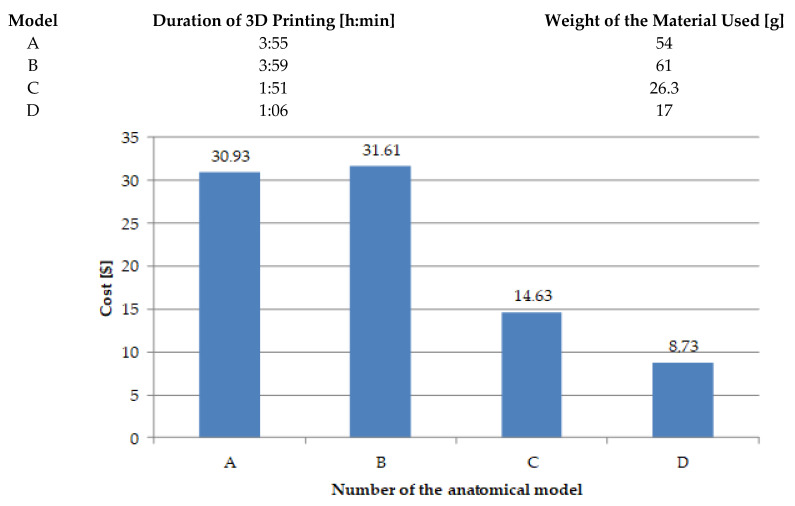
The amount of material used and the costs of manufacturing the appropriate anatomical models in the FFF.

Figure 14 presents the times, the amount of material used, and the costs of manufacturing the appropriate anatomical models in the fused filament fabrication (FFF) additive technology. The valuation of the implementation of a specific model must consider the specificity of the applied additive technology, production time with this technology, and fixed costs. The costs were calculated assuming that the material cost of the models is 25.17 ($). The operating time of the machine was also included in the fixed costs. Such a low cost of making models of anatomical structures is possible if the method mentioned earlier, material, and equipment are used, the low depreciation cost. In the case of industrial devices working in a similar method or other additive technologies, the operating cost is much higher (a critical element of the price is the depreciation of the machine). All this is related to the price of the device and the cost of the material used to make the model. As an example, we can give the implementation of the anatomical model described as “A” in the selective laser sintering (SLS) additive technology, where the cost of production increases almost threefold and amounts to about 87.11 ($).

In the case of the analysed accuracy of models, the deviation of shape fell within the tolerance range of +/− 0.15 mm. Taking into account the current recommendations, the required accuracy of the printout for models used in the treatment planning process should be in the range of +/− 0.25 mm [78,79]. Thanks to the use of two control processes in the procedure (which allow for assessment of the accuracy of the models and the surgical process), it is possible to continuously improve the process in terms of the controlled preparation of templates and surgical tools in terms of the accuracy expected during the procedures. It is important that the procedure for controlling the accuracy of a surgical procedure gives valuable information when determining the correct rehabilitation process.

The main purpose of the presented procedure is to increase the precision of the surgery. The mandible is the most specific bone structure in the stomatognathic system. It is one moving bone subjected to multidirectional dynamic loads in the masticatory organ [31]. Apart from functional tasks, supporting the tissues of the lower face and the floor of the mouth is also important. As a result of breaking the mandibular continuity, the suprahyoid muscles, devoid of their mandibular attachment, collapse, causing airway obstruction. On the other hand, the mandibular stumps, left without anastomosis after resection, move upwards and medially because of the action of the pterygoid muscles, temporal muscles, and masseter muscles. The above changes lead to impaired airway patency, dysphagia, speech and chewing disorders, and distortion of the lower part of the face [80,81]. The problems mentioned above may disrupt the proper functioning of the stomatognathic system, the masticatory system, and the aesthetic functions of the face. So far, when supplementing the restored fragment of the mandibular bone with the fibula, the surgeon during the procedure, on the basis of his experience and spatial imagination, decided where and at what angle to carry out the cut of the fibula so that the bone fragment could be used to obtain a shape similar to the mandible. By carrying out these stages before the procedure on the basis of printed models, the accuracy of adherence of bone fragments during the surgical procedure was increased, and thus, more favourable conditions for their fusion were ensured [82]. The aesthetic effects of the reconstruction were also improved. Due to the use of modern methods of reconstructive engineering, CAD, and 3D printing, the treatment time has been reduced by about 20% [83]. It was connected with less blood loss during surgery, shorter time of ischemia of the transplanted flap, fewer complications in the circulation of the flap, and shorter convalescence [84]. Despite the additional costs of the applied technology, thanks to the above-mentioned advantages, reductions in the total cost of treatment have been shown. Even though in the course of the research on 14 patients no detailed statistical studies were carried out on, among other things, shortening the time of surgery and blood loss, the observations made themselves are consistent with those presented in the publications [82,83,84]. Other advantages of the procedure include: the reduction of operator stress, and improved communication between members of the tumour resection team and doctors responsible for reconstruction. In addition, the cost of preparing templates used to train doctors is lower compared to animal or human preparations. Time is an important factor in the process of virtual planning, template modelling, and printing. Based on the literature data, it is estimated that the average time needed to apply the technology is approximately 14 days, which is not without significance in the case of malignant neoplasms, as the total treatment time should not be more than 100 days [82]. Moreover, it is necessary to purchase software and hardware, spend a certain amount of time learning how to use them, and implement procedures (bearing the costs of the material and labour of the printer operator). Thanks to the cooperation agreement concluded in 2018 between the Fryderyk Chopin Provincial Clinical Hospital No. 1 in Rzeszów–Maxillofacial Surgery Clinic, and the Rzeszów University of Technology, communication between employees of both units was improved, which significantly shortened the time of planning and performing the surgery. Currently, in the case of patients requiring rapid intervention, the average time from CT examination to model making is about 7 days. Another effect of cooperation is the obtained patent “A method of producing anatomical models”, which describes the entire procedure presented in the article. The greatest challenge currently faced by the research team is to create the possibility of a quick assessment of the scope of the surgery. The cases when the resected area should be widened are very common. It is not always possible to precisely define the boundary between healthy and diseased tissues based on the visual assessment of the imaging data [84]. At present, the research team prevents such situations by means of close cooperation between the person planning the procedure in virtual space and the surgeon. However, discussions on the estimation of a sufficient resection area often take a lot of time. With this in mind, it is planned to further expand the presented procedure by using the deep learning method in a more accurate assessment of the disease area [85,86]. These methods will improve communication between members of the research team, which will further reduce the time needed to prepare models for surgery.

## 5. Conclusions

Designing and making models for a surgical procedure is not a simple task. This is especially true of the area the facial part of the skull, which consists of bone tissues of very complex geometry. Significant knowledge and skills in the field of medicine and technical sciences are needed, which will allow the full use of currently available tools in the processes related to the reconstruction of the facial parts of the skull. This is especially true of the mandible, which is the only movable bone in this area. Knowledge of the procedure presented in the article is a crucial support in the field of controlled preparation of templates and surgical tools in terms of the accuracy expected during the procedures. Despite the additional costs of the applied technology, reductions in the total cost of treatment have been achieved. Based on the results presented in the publication, further studies will be carried out on a broader group of patients. They will be concerned with accurate statistical tests assessing the time of the surgical procedure and hospital stay and the amount of blood loss. Additionally, further development of the procedure is also planned, especially at the stage of numerical processing of DICOM data, by using the deep learning method in segmentation. These methods should allow for a further reduction of the time needed for preparation of models and a more precise definition of the resection area.

## 6. Patents

Resulting from the presented procedure in this manuscript is the granting of a patent “A method of producing anatomical models” Currently, it is waiting for a number to be issued, which will take place at the end of the calendar year.

## Data Availability

The data presented in this study are available on request from the corresponding author.
